# Celastrol Suppresses Tumor Cell Growth through Targeting an AR-ERG-NF-κB Pathway in TMPRSS2/ERG Fusion Gene Expressing Prostate Cancer

**DOI:** 10.1371/journal.pone.0058391

**Published:** 2013-03-06

**Authors:** Longjiang Shao, Zhansong Zhou, Yi Cai, Patricia Castro, Olga Dakhov, Ping Shi, Yaoxia Bai, Huixiang Ji, Wenhao Shen, Jianghua Wang

**Affiliations:** 1 Department of Pathology and Immunology, Baylor College of Medicine and Michael E. DeBakey Department of Veterans Affairs Medical Center, Houston, Texas, United States of America; 2 Department of Urology, South West Hospital, Chongqing, People's Republic of China; Florida International University, United States of America

## Abstract

The TMPRSS2/ERG (T/E) fusion gene is present in the majority of all prostate cancers (PCa). We have shown previously that NF-kB signaling is highly activated in these T/E fusion expressing cells via phosphorylation of NF-kB p65 Ser536 (p536). We therefore hypothesize that targeting NF-kB signaling may be an efficacious approach for the subgroup of PCas that carry T/E fusions. Celastrol is a well known NF-kB inhibitor, and thus may inhibit T/E fusion expressing PCa cell growth. We therefore evaluated Celastrol’s effects *in vitro* and *in vivo* in VCaP cells, which express the T/E fusion gene. VCaP cells were treated with different concentrations of Celastrol and growth inhibition and target expression were evaluated. To test its ability to inhibit growth *in vivo*, 0.5 mg/kg Celastrol was used to treat mice bearing subcutaneous VCaP xenograft tumors. Our results show Celastrol can significantly inhibit the growth of T/E fusion expressing PCa cells both *in vitro* and *in vivo* through targeting three critical signaling pathways: AR, ERG and NF-kB in these cells. When mice received 0.5 mg/kg Celastrol for 4 times/week, significant growth inhibition was seen with no obvious toxicity or significant weight loss. Therefore, Celastrol is a promising candidate drug for T/E fusion expressing PCa. Our findings provide a novel strategy for the targeted therapy which may benefit the more than half of PCa patients who have T/E fusion expressing PCas.

## Introduction

Prostate cancer (PCa) is a heterogeneous disease which is still poorly understood. The pathways altered at high frequency in specific patient tumor types need to be better defined before designing individually targeted therapy. Encouragingly, over the past a few years important progress has been made in the subclassification of PCa, in particular the finding that the TMPRSS2/ERG (T/E) fusion gene is present in the majority of PCas and is thus the most common genetic lesion discovered in PCa [1], [2], [3]. Many studies have consistently shown that the T/E fusion gene can promote PCa invasion and to a lesser extent proliferation and decrease differentiation [4], [5]. The high frequency of this alteration and its important role in PCa tumor biology makes it an outstanding therapeutic target in PCa. We have shown that stable shRNA expression that specifically targets T/E fusion transcripts significantly decreases tumor growth *in vivo*, but they do not completely eliminate it [6], indicating the need for the combination therapy which can overcome the resistance and/or weak response to the single targeted treatment. Our studies show that NF-kB signaling, particularly phosphorylation of NF-kB p65 Ser536 (p536) is highly activated in these T/E fusion expressing cells via ERG [7]. NF-kB signaling has been shown previously to play an important role in PCa growth, angiogenesis, tumorigenesis and metastatic progression [8], [9], [10], [11], [12]. Given the pleiotropic effects of NF-kB on tumor progression, we hypothesize that targeting NF-kB signaling may be an efficacious approach for the subgroup of PCas who carry T/E fusions.

In recent years, there is a growing interest in identification of potent and bioactive molecules from natural sources including traditional Chinese medicine [13], [14]. Some of these molecules may offer patients safer long term therapeutic options [15]. Celastrol, a pharmacologically active compound from the extract of the Chinese Thunder God Vine which has been used in China for hundreds of years, has attracted great attention recently due to its significant anti-inflammatory and anti-cancer activities [16], [17]. Many studies have demonstrated that Celastrol can modulate multiple signaling pathways involved in disease and therefore exhibit potential therapeutic value against various chronic diseases and cancers [16], [17]. Celastrol has been shown to inhibit the growth of many types of cancer cells and suppress tumor initiation, progression and metastasis in various animal models *in vivo*, including breast cancer [18], lung cancer [19], pancreatic cancer [20], glioma [21], and melanoma [19] as well as PCa [22], [23], [24], and so far no significant adverse effects have been reported. Many important molecular targets of Celastrol have been identified, including NF-kB, Hsp90, the proteasome, VEGFR, AKT/mTOR, c-Jun and others [16], [18], [22], [25], [26], [27], but inhibition of NF-kB pathway appears to account for much of its therapeutic effects.

Despite the high frequency of T/E fusion detected in PCa patients, only one commonly used PCa cell line (VCaP) endogenously expresses T/E fusion. It is an androgen dependant cell line and expresses high level of AR, which can drive expression of ERG under the control of the AR dependent TMPRSS2 promoter. It also shows high level of p536 compared with almost undetectable expression in other commonly used PCa cell lines, including LNCaP, PC3 and DU145 [7]. We therefore used VCaP in our studies. Here, we show that Celastrol is a potent p536 inhibitor which can significantly inhibit VCaP cell growth both *in vitro* and *in vivo*. In addition, our data revealed a novel signaling cascade of AR-ERG-p536 targeted by Celastrol. The targeting of AR-ERG-NF-kB by Celastrol is novel and is seen even when T/E fusion expressing PCa cells are exposed to very low concentrations of Celastrol. Under such conditions, the other reported major pathways remain unaffected. Based on all evidence and its potent biologic effects, Celastrol may have potential clinic use for patients who carry T/E fusion in their PCa tumors.

## Materials and Methods

### Cell Culture

VCaP cells were maintained in the DMEM (high glucose) with 10% fetal bovine serum (FBS). VCaP-Luc cells expressing luciferase used for live animal imaging were maintained in the same DMEM medium but with 2 µg/ml puromycin [6]. LNCaP, PC3 and DU145 were cultured in RPMI with 10% FBS.

### Western Blotting

ERG anti-rabbit antibody (1∶2000) was obtained from Epitomics, Inc (Burlingame, CA, USA, cat# 2805-1 ). AR-V7 (AR3) antibody was purchased from A&G Precision Antibody™ (Columbia, MD, USA, cat# AG10008) and used as 1∶1000 dilution. Anti-p65, phospho-p65 Ser536, AR, IKBα, HSP90, total-AKT and phospho-AKT Ser473 were all obtained from Cell Signaling Technology, Inc (Danvers, MA, USA) and were used at 1∶1000 dilution for Western blotting using procedures described previously [28]. Anti-β-actin control was performed as described previously [28]. Blot signals were visualized using enhanced chemiluminescence (Thermo Fisher Scientific, Inc) and exposed and developed with films or Bio-Rad imaging System and quantified by a densitometer using Quantity One (Version 4.5.2, Bio-Rad Laboratories, Inc, Hercules, CA ).

### Quantitative Real-time PCR

T/E fusion and β-actin primers were as described previously [3]. 5 µl of the template cDNA (1∶20 dilution) were used in a final reaction volume of 15 ul. The Master mix for real time PCR contained 2 mM MgCl2, 0.4 µM each forward and reverse primers and 7.5 µl of DNA Master SYBR GREEN (2×; Applied Biosystems Inc, Foster City, CA, USA). Real-time PCR was done by using the ABI instrument from followed by a 3-step PCR protocol with different annealing temperature shown below. Primers for AR, AR3, p65 and CCL2 were: AR RTF: 5′-CTACTCCGGACCTTACGGGGACATGCG-3′; AR RTR: 5′-GGGCTGACATTCATAGCCTTCAATGTGTGAC-3′; AR3 RTF: 5′-CTACTCCGGACCTTACGGGGACATGCG-3′; AR3 RTR: 5′-TGCCAACCCGGAATTTTTCTCCC-3′; P65 RTF: 5′-CTGCAGTTTGATGATGAAGA-3′; P65 RTR: 5′-TAGGCGAGTTATAGCCTCAG-3′; CCL2 RTF: 5'- TCTCAGTGCAGAGGCTCG -3'; CCL2 RTR: 5'- GTTTGGGTTTGCTTGTCCAG -3'. The relative expression by ΔCt among different experimental groups was collected and normalized to β-actin expressions.

### Proliferation Assay

1×10^5^ cells were seeded on 24-well plates in triplicate and attached cells were counted using a cell counter as described previously [29]. Celastrol was purchased from Sigma-Aldrich Co. LLC. (Cat# C0869) and dissolved in DMSO as recommended. For VCaP cells Celastrol treatment was started 48 h after seeding. LNCaP, PC3 and DU145 cells were treated with Celastrol 24 h after initial seeding. Different doses of Celastrol were applied to each experimental group. The experiment was repeated three times.

### VCaP-Luc Subcutaneous Mice Model

Twelve-week old nude mice were used. 4×10^6^ VCaP cells expressing luciferase (VCaP- Luc cells) were mixed with 100 ul matrigel (BD Bioscience, San Jose, CA, USA) in a total volume of 200 ul and injected subcutaneously. Tumor growth was monitored weekly after initial injection using IVIS imaging system (Xenogen, Alameda, CA, USA) [6]. Sixteen mice showed a 1×10^5^ luminance reading one week after injection and were included for the treatment experiments. They were randomly separated into 2 experimental groups of 8 mice in each group. Mice in the treatment group were injected intraperitoneally (I.P) with 200 ul of 0.5 mg/kg Celastrol dissolved in 3%DMSO and PBS, while the control animals received 200 ul of vehicle (3%DMSO and PBS). The treatment was done 4 times per week for 3 weeks. Mouse weights were monitored twice weekly. Mice were euthanized 24 hours after the final injection and primary tumors were excised, weighed, and a portion of the tumor was frozen in liquid nitrogen for molecular analysis and another portion fixed and paraffin-embedded. Necropsy was also performed on mice to rule out side effects of treatment. Differences in mean tumor size are examined by t-test. Protein and RNA extracts were prepared for further analysis. A tissue microarray (TMA) was prepared for IHC analysis. All procedures were approved by the Baylor College of Medicine Institutional Animal Use and Care Committee (IACUC protocol number #AN-4542).

### *Immunohistochemistry* and Analysis of Apoptosis

Immunohistochemistry (IHC) of VCaP subcutaneous tumors was performed using anti-phospho-p65-Ser536 and ERG as described previously [7], [29]. A mouse monoclonal anti-AR from Biocare Medical, Concord, CA, USA (Cat# CM109) was used for IHC to assess AR expression in tumor sections using 1∶50 dilution. Slides were photographed using a Nikon Eclipse E400 microscope connected with Nuance Multispectral Imaging System at 40× or 200× magnifications with 3.3 megapixel resolution. For Ki-67 and CD31 IHC and TUNEL, images were saved as JPEG files with 4–6 images were taken for each slide, covering the entire tumor area. The numerical value for percent stained (PS) is determined by using Image J software (http://rsb.info.nih.gov/ij/) and compared using t-tests.

## Results

### Celastrol is a Potent p536 Inhibitor

**Figure 1:**
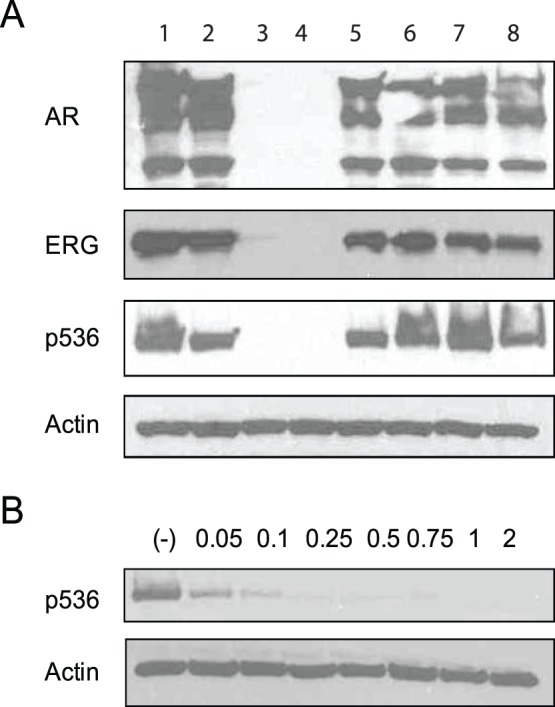
Celastrol is a potent p536 inhibitor. (**A**)VCaP cells were treated with different NF-κB inhibitors for 18 h. Western blot shows that Celastrol 2 µM for 18 h treatment can significantly inhibit p536 expression while 481407 (2 µM) and MG132 (2.5 µM) almost had no impact on p536 expression. In addition to p536, dramatically decreased AR and ERG expression at protein levels were seen in Celastrol treated group, but they are not decreased by other two inhibitors. Duplicate wells are shown for each group. β-actin was used as control. (**B**) VCaP cells were treated with different concentration of celastrol for 2 h. Significant decreased p536 expression was seen in all groups by western blot, including the lowest concentration group of 0.05 µM, strongly suggesting Celastrol is a potent p536 inhibitor. β-actin was the control.

Given the finding that NF-κB signaling is highly activated in T/E fusion expressing cells, we sought to test the hypothesis that targeting NF-κB signaling may be a viable therapeutic approach for T/E fusion expressing PCa. Therefore, we evaluated several candidate NF-κB inhibitors including two NF-κB activation inhibitors (481407 compound and Celastrol) and MG132, a NF-κB inhibitor functioning at the level of proteasome inhibition [9], [30]. As shown in Fig. 1A, Celastrol can significantly abolish p536 in VCaP cells when used at concentration of 2 µM for 18 h, while the other two NF-κB inhibitors 481407 (2 µM) and MG132 (2.5 µM) [9] showed no such effect, as did PS1145 (data not shown). The inhibition of p536 expression by Celastrol was evident even with a 2 h treatment at 0.05 µM Celastrol resulting in a greater than 60% reduction of p536 expression (Fig. 1B), indicating Celastrol is a potent p536 inhibitor.

It has been reported that Celastrol can inhibit AR expression in LNCaP cells [22]. If it targets AR in VCaP cells it could cause downregulated ERG expression which in turn could result in decreased p536 expression as described previously by our group [7]. As shown in Fig. 1A, Celastrol at 2 µM for 18 h can significantly inhibit AR and ERG expression in addition to totally abolishing p536 expression in VCaP cells, while the other two inhibitors showed no such effects.

### AR-ERG-p536, a Novel Signaling Cascade Targeted by Celastrol in vitro

**Figure 2:**
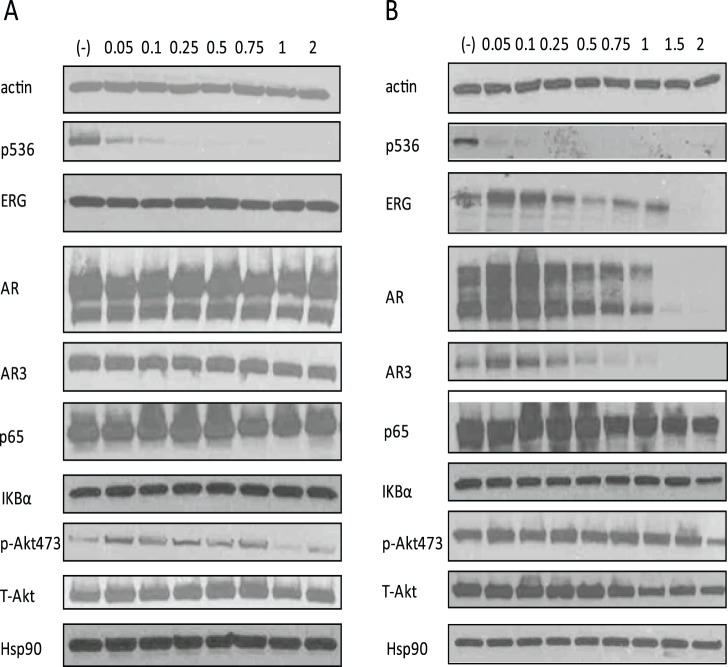
Celastrol targets AR-ERG-p536 in T/E fusion expressing PCa cells at protein level. (**A**) Western blot shows that Celastrol 2 h treament can significantly target the p536 expression in VCaP cells. Celastrol concentrations are shown on the top of each lane. Short time treatment of Celastrol does not affect AR, AR3, ERG expression, and total p65, IKBα as well as other well known Celastrol targets such as total AKT, phosphorylated AKT-473 and Hsp90. (**B**) When Celastrol treatments last for 24 h, p536 signaling was almost abolished in all groups. In groups in which Celastrol concentration is equal or higher than 0.5 µM, significant decreased AR and ERG as well as AR3 expression were seen. When concentration reaches 2 µM, 24 h treatment can affect multiple signaling such as phospho-AKT. β-actin was used as the control.

**Figure 3:**
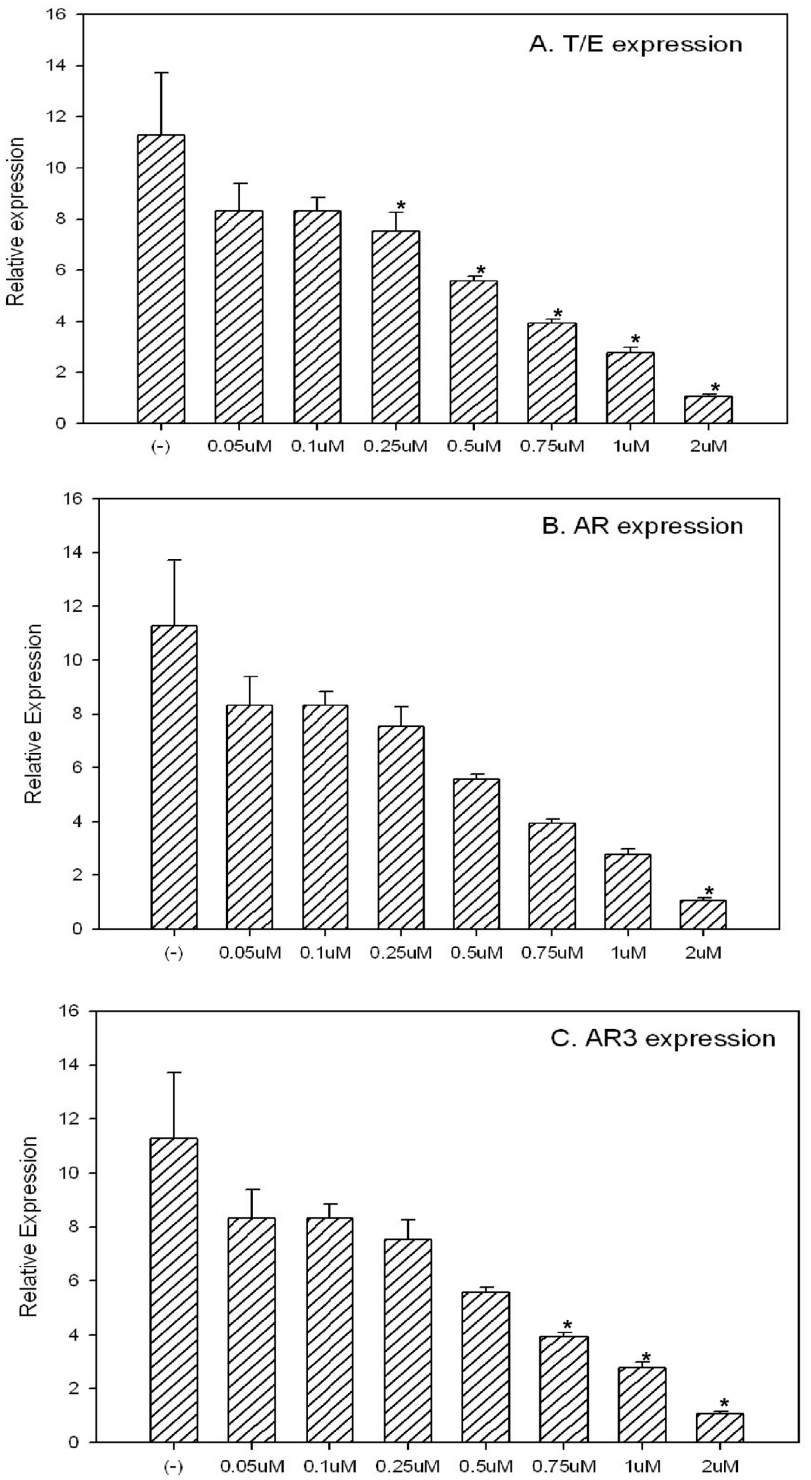
Celastrol targets T/E fusion, AR and AR3 expression at transcriptional level by quantitative Real-time PCR. When VCaP cells were treated with different concentration of Celastrol for 24 h, inhibition of targeted genes were evaluated by quantitative real-time PCR. β-actin was used as the control gene. T/E fusion expression(**A**), AR(**B**) and AR3 (**C**) expression are shown in each figure. The inhibition of these genes at RNA levels by Celastrol are significant in a dose-depedent manner. Asterisks indicate statistically significant differences between Celastrol treated group and the control by t-test.

There were many other targets of Celastrol reported in different system/cells, including the AKT pathway and Hsp90. In order to prove the inhibition of AR-ERG-p536 is unique in T/E fusion expressing cells, we treated VCaP cells with Celastrol for 2 h and 24 h using different concentrations ranging from 0.05 µM to 2 µM. As shown in Fig. 2A, Celastrol treatment for 2 h can inhibit p536 expression significantly even at the very low concentration of 0.05 µM, but this concentration showed almost no effects on AR (including AR3, an active isoform of AR) or ERG expression as well as other proteins including total p65, IKBα, total-AKT, phospho-AKT 473 and Hsp90. In a 24 h treatment experiment, the lower concentration of 0.5–1 µM, Celastrol can significantly inhibit AR, AR3andERG protein expression in addition to p536 expression, while p65, IKBα, phospho-Akt473, total-Akt and Hsp90 are almost not affected. Due to its pleiotropic biologic activities, caution should be taken in terms of the dose of Celastrol used for *in vitro* or *in vivo*. At higher concentration, multiple important pathways may be affected since 2 µM Celastrol starts to down regulate phospho-AKT473 (Fig. 2B). We also found that only 0.75 µM Celastrol for 24 h is required to abolish p-AKT473, total AKT and total AR expression in LNCaP cells (data not shown) while the previous study used 5 µM to show AR suppression and other biologic effects caused by Celastrol in the same cell line [22]. Therefore, defining the minimum concentration/dosage is critical to avoid any potential toxicity caused by inhibiting multiple affected pathways. Our *in vitro* data indicates that Celastrol, when used between 0.5 µM to 1 µM, can target AR-ERG-p536 signaling cascade almost exclusively without affecting other major pathways. We further tested ERG, AR and AR3 expression at RNA level in these Celastrol treated cells by quantitative RT-PCR [3]. As shown in Fig. 3, we confirmed the inhibition of T/E fusion, AR and AR3 gene expression by Celastrol at the RNA level in a dose-dependent manner.

### Celastrol can Inhibit CCL2 Expression at Both the RNA and Protein Level

**Figure 4:**
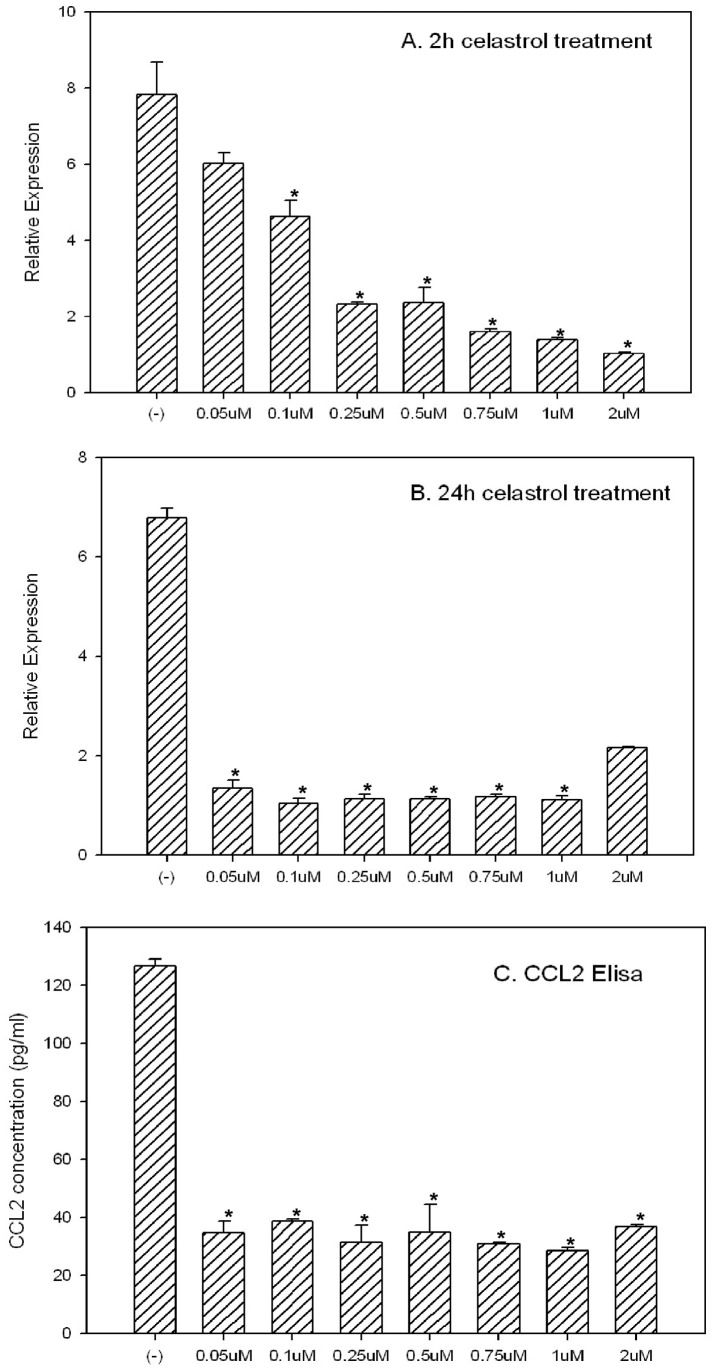
Celastrol can significantly inhibit CCL2 expression both at transcriptional and protein level. (**A**) VCaP cells were treated with Celastrol for 2 h, the inhibition of CCL2 at RNA level was shown in a dose-depedent manner by real-time PCR; (**B**) when Celastrol treatments were done for 24 h, even 0.05 µM can dramatically decrease CCL2 expression; (**C**) when exposed to Celastrol for 24 h, secreted CCL2 protein in culture medium by VCaP cells are significantly decreased. ELISA data are shown. Each concentration was tested in triplicate and the experiment was repeated three times. Asterisks indicate statistically significant differences between Celastrol treated group and the control by t-test.

As we reported earlier one candidate gene, CCL2, is upregulated in T/E fusion expressing PNT1a cells [7]. This upregulation of CCL2 could result from the elevated NF-κB signal in these cells by the T/E fusion. CCL2 (also called MCP-1) is a chemokine which is a potent regulator of PCa cell migration and proliferation [31], [32], [33]. CCL2 is expressed by endothelial cells within the tumor microenvironment but can also be expressed by tumor cells directly [31], [32]. Most importantly, emerging evidence suggests CCL2 is a direct transcriptional target of NF-ΚB [34]. Based on this evidence, we hypothesized that Celastrol may have a direct impact on CCL2 expression. We treated VCaP cells with different concentrations of Celastrol for 2 h or 24 h. As shown in Fig. 4A, Celastrol treatment for only 2 h can significantly decrease CCL2 mRNA expression in VCaP cells as assessed by Q-RT-PCR and such inhibition is dose-dependent. If treatments last for 24 h, even 0.05 µM Celastrol can significantly decrease the CCL2 mRNA expression (Fig. 4B). Of note, other studies have shown CCL2 can be secreted into culture medium by VCaP cells [31]. We therefore collected culture medium from each Celastrol treatment group and test CCL2 concentration using CCL2 Elisa kit from ebioscience.com. As shown in Fig. 4C, all Celastrol treatment groups (24 h treatment) including the lowest concentration treatment group of 0.05 µM showed significantly decreased CCL2 in the culture medium. Therefore, we have additional evidence that Celastrol, as a potent NF-κB inhibitor, can significantly affect the downstream targets of NF-κB. However, whether phosphorylation of ser536 plays a role in regulating CCL2 expression is currently unknown.

### Celastrol can Significantly Inhibit T/E Fusion Expressing VCaP Cell Growth in vitro

**Figure 5:**
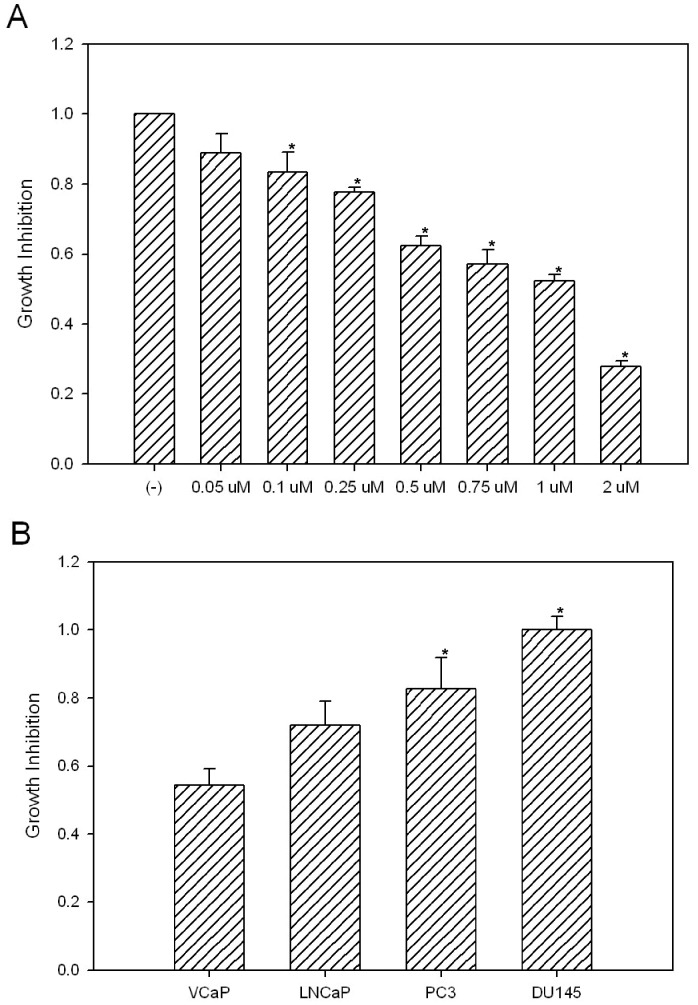
Celastrol inhibit VCaP cell growth *in vitro*. (**A**) Cells (1×10^5^) were plated in 35 mm dishes in complete medium and treated with different concentration of Celastrol for 24 h. Proliferation of the 8 groups of VCaP cells were measured using a Coulter counter. Cells were trypsinized and counted in triplicate. Cell numbers for each group are divided by the total cell number in the control group without Celastrol treatment. The experiment was repeated three times. Mean +/− standard deviation is shown. Asterisks indicate statistically significant differences between Celastrol treated group and the control. (**B**) 1×10^5^ of VCaP, LNCaP, PC3 and DU145 were exposured to 0.5 µM Celastrol for 24 h, cell numbers were counted for each group and divided by the total cell numbers in control groups to obtain the survival ratio.The experiment was repeated three times. Asterisks indicate statistically significant differences between different cell lines and the VCaP cells.

Celastrol can significantly inhibit PCa cell growth *in vitro* including most commonly used PCa cell lines of LNCaP, PC3 and DU145 [22], [35]. However, Celastrol has never been tested on VCaP cells. Since Celastrol can target AR-ERG-NF-κB, three major pathways in VCaP cells, we reasoned that it is likely to exhibit strong inhibition of VCaP cell growth. We treated VCaP cells with different concentrations of Celastrol for 24 h and cell numbers were counted using a cell counter. As expected, VCaP cell growth can be significantly inhibited by Celastrol in a dose-dependent manner (Fig. 5A). Since VCaP cells express high level of p536 (compared to almost undetectable signal from other PCa cells), and AR, we hypothesize that T/E fusion expressing VCaP cells would be the most sensitive PCa cell line to Celastrol treatment while LNCaP would be sensitive due to AR expression and the AR negative PC3 and DU145 cells should be relatively insensitive. To test this hypothesis, we used 0.5 µM Celastrol, the lowest effective dose which shows significant impact on AR, ERG, and p536 expression (see *in vitro* data in Fig. 2B), to treat LNCaP, PC3, DU145 and VCaP cells for 24 h. Under our experimental condition, the cell survival ratios are 55%, 72%, 83% and 100% for VCaP, LNCaP, PC3 and DU145 respectively (Fig. 5B). This data again strongly suggests Celastrol may be a good candidate drug for T/E fusion expressing PCa.

### Celastrol can Inhibit T/E Fusion Expressing VCaP Cell Growth in vivo

**Figure 6:**
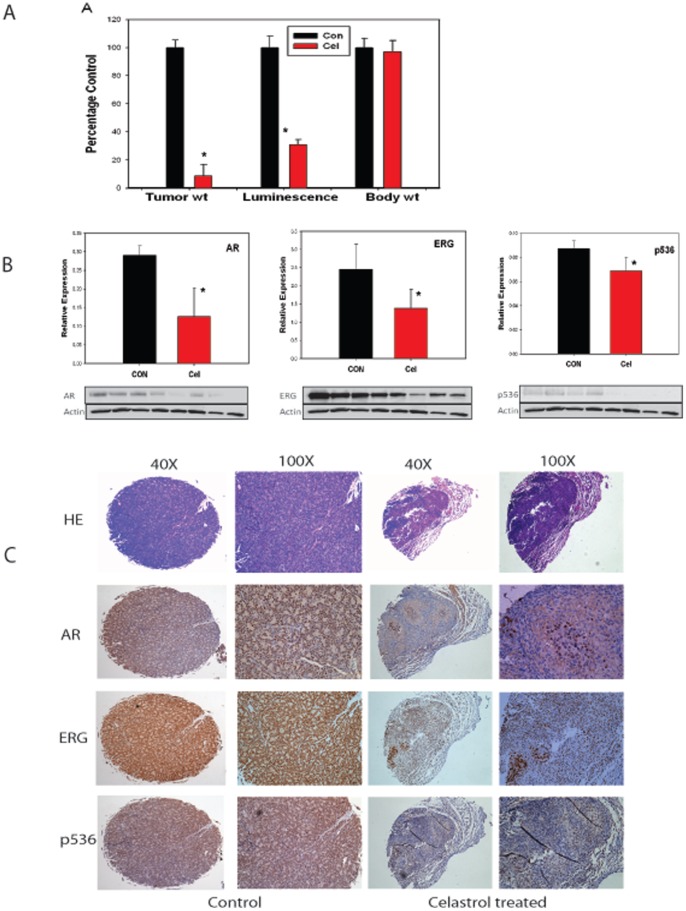
Celastrol inhibit VCaP cell growth *in vivo*. (**A**) Nude mice were injected subcutaneously with VCaP cells expressing luciferase. After one week mice were treated with 0.5 mg/kg Celastrol or vehicle only. Luciferase flux of tumors prior to euthanasia and tumor and body weights at termination of treatment are shown. Asterisks indicate statistically significant differences between treated group and control group. (**B**) Inhibition of AR, ERG and p536 by quantitative Western blot normalized to β-actin. Representative Western blot of VCaP tumor extracts with antibodies against AR, ERG or p536 is shown. In each western blot, lanes 1–4 are the control tumors and lanes 5–8 are Celastrol treated tumors. β-actin is a loading control. (**C**) A tissue microarray (TMA) was prepared for IHC analysis for these mice tumors collected. Representative samples are shown. Images in the left two panels are for a control mouse tumor and images in the right two panels are for a Celastrol treated mouse tumor. H&E, AR, ERG and p536 staining are shown both at magnification of 40× and 100×. Dramatically decreased AR, ERG and p536 expression caused by Celastrol treatment are seen.

Based on these *in vitro* results, we moved forward to *in vivo* experiments using Celastrol to treat VCaP tumors in a subcutaneous tumor model. One week after subcutaneous injection of 4×10^6^ luciferase-expressing VCaP cells into nude mice, Celastrol treatment was initiated. Sixteen mice showed more than 1×10^5^ luminance reading one week after injection and were included in for the experiments. Mice in the treatment group were injected with 0.5 mg/kg Celastrol I.P. The treatment was done 4 times per week for 3 weeks. Mice were weighed and luciferase based tumor imaging was performed weekly. Mice were euthanized 24 hours after the last injection and primary tumors excised, weighed and snap frozen for molecular studies or submitted for histopathology and a complete necropsy performed on mice. No significantly decreased body weight was noted (Fig. 6A). One mouse in Celastrol treatment group showed peritoneal inflammation but no significant toxicities were observed in the lung, kidney, liver, spleen and heart. We observed that some tumors started shrinking after 2 weeks of Celastrol treatment and by the end of the experiments four mice showed luminescence lower than 1×10^5^. Luciferase activity prior to euthanasia is shown in Fig. 6A. There was a marked inhibition of tumor growth in Celastrol treated group and at the end of treatment tumor luminescence was decreased ∼70% (p = 0.048, t-test) compared to the control group. We were able to collect 5 tumors from the Celastrol treated group and 7 tumors for the control group. Final tumor weight in treatment group was decreased to only ∼10% of that in untreated group (P = 0.0034, t-test). Thus 0.5 mg/kg Celastrol treatment can significantly inhibit VCaP tumor growth *in vivo*.

Analysis of tumors collected from mice treated or untreated with Celastrol was performed using Ki67 IHC followed by quantitative image analysis and showed no significant difference. TUNEL staining showed that there were more apoptotic cells found in Celastrol treated tumors compared with untreated tumors. The difference was significant (P<0.05, t-test). Thus the decreased tumor growth can be attributed to the increased cell death, which is consistent with previous reports [22], [36]. We also performed anti-CD31 IHC to evaluate microvessel density as a marker of angiogenesis. We observed no significant difference between treated and untreated tumors.

We have shown *in vitro* that Celastrol can target AR, ERG and p536. We therefore sought to determine if these same targets were affected *in vivo*. Due to very small tumor mass collected in Celastrol treated group (the average weight of these 5 tumors were 0.0345 g), we could only have 4 tumor samples for western analysis. Therefore, we randomly chose 4 tumors from control group to compare the expression at the protein level. As shown in Figure 6B, significantly decreased AR, ERG and p536 expression were seen in Celastrol treated group compared to control group (P<0.05, t-test). We also observed slightly decreased Hsp90 expression in treated tumors, but the difference was not significant (data not shown, p = 0.1, t-test). In addition, we found that Celastrol has no affect on total AKT expression. Due to very limited tumor mass, we were not able to obtain AR3 and phospho-AKT expression in these tumor samples.

Overall, the data indicates Celastrol can significantly inhibit VCaP cell growth *in vivo* by targeting AR-ERG-NF-κB signal and suggests Celastrol may have therapeutic potential for T/E fusion carrying PCa.

## Discussion

Significant progress has been made in recent years regarding the subclassification of PCa based on the genetic alterations in the tumor cells, including chromosomal rearrangements involving ETS family transcription factors or overexpression of SPINK1, a gene encoding a secreted serine protease inhibitor. Encouragingly, Ateeq et al. provided evidence supporting a rationale for targeting the SPINK1 in SPINK1^+^/ETS^−^ PCas and demonstrated that combined monoclonal antibody treatment targeting both SPINK1 and epidermal growth factor receptor can cause more significant reduction in tumor formation than either monoclonal antibody alone, providing a strong evidence for targeted therapy for SPINK1^+^/ETS^−^ PCas [37]. The impact of this finding is limited by the finding that SPINK1 is only expressed in a small percentage of PCas (∼10%) and is not seen in ETS-factor expressing PCa. The majority of ETS-positive PCas carry T/E fusions which are present in more than half of PCas. Thus there is an urgent need to discover novel treatment options for this major subgroup of PCas. We have shown previously that targeting T/E fusion transcripts using shRNA can significantly decrease tumor growth *in vivo* [6], but they do not completely eliminate it. We have also shown that NF-κB signaling, via phosphorylation of NF-κB p65 Ser536 is highly activated in these T/E fusion expressing PCa cells (>90% of total case analyzed) and the absence of p536 is associated with decreased biochemical recurrence [7]. Given the pleiotropic effects of NF-κB on tumor progression, these findings suggest that NF-κB signaling, particularly the phosphorylation of p65 Ser536, plays a critical role in tumorigenesis in PCas bearing T/E fusions. Therefore, we hypothesize that a two-step personalized therapy strategy of targeting both T/E fusion and NF-κB signaling, which is similar to the approach for SPINK1^+^/ETS^−^ PCas as discussed previously [38], may be a viable approach for this subgroup of PCa who carries T/E fusion.

In this study, we show that Celastrol, a well known NF-κB inhibitor, may be an excellent candidate drug for treating T/E^+^/p536^+^ PCa. Celastrol can modulate multiple signaling pathways involved in cancers and therefore can significantly inhibit cancer cell growth [16], [17]. Multiple molecular targets of Celastrol have been identified including AKT, Hsp90 and others [16], [18], [22], [25], [26], [27], [39]. We show first that Celastrol is a potent p536 inhibitor both *in vitro* and *in vivo*. Very low concentration of 0.05 µM Celastrol treatment for 2 h significantly decreased p536 expression in VCaP cells. Similarly, 0.5 mg/kg Celastrol treatment 4 times/week, the lowest dose ever reported in literature, can significantly decrease the p536 levels in VCaP xenograft tumors. Phosphorylation of p65 has been shown by many groups to enhance p65 transcriptional activity (see reviews [40] and [41]), but the exact role of p536 in PCa cells especially in T/E fusion expressing cells is unknown and the genes specifically regulated by p536 have never been explored in PCa. Our data shows that the expression of CCL2, a well known target of NF-κB p65 and a potent regulator for PCa cell migration and proliferation [31], [32], [33], can be dramatically inhibited by Celastrol at both mRNA and protein level, which provides further evidence suggesting that Celastrol may have strong biological effects on the T/E fusion expressing PCa cell growth and/or invasion by targeting downstream targets of NF-κB signal. It is very possible that phosphorylation of Ser536 plays a critical role in regulating CCL2 expression. These questions are currently under investigation in our laboratory. We observed that 0.05 µM celastrol treatment for 24 h can specifically inhibit Ser563 phosphorylation and CCL2 expression, while they have no significant effect on VCaP cell proliferation (Fig. 5A). One possible explanation could be that Celastrol treatment, at lower concentration level for short time period, did not diminish p536 and CCL2 expression completely. The continued CCL2 expression may be still above a control threshold which is enough to maintain cell growth. An alternative explanation is that lower concentration of Celastrol might trigger other signaling pathways involved in cell growth regulation, which potentially masks the phenotypic effects of decreased p536 and CCL2 expression.

In addition to the decreased p536 expression, we discovered that Celastrol can target AR and ERG signaling in VCaP cells. AR, ERG and NF-κB are three major pathways involved in promoting tumorigenesis of PCa. Targeting each pathway showed significant inhibition on tumor growth. The fact that Celastrol has potent effects on AR expression, which can result in downregulated ERG expression followed by decreased NF-κB activities, further highlights Celastrol’s antitumor action and potential therapeutic value. Under our experimental condition, when lower doses of Celastrol (0.5–1 µM) were used *in vitro*, AR-ERG-p536 signaling is almost exclusively inhibited in VCaP cells while other reported major pathways remain unaffected. When treated with higher concentrations of Celastrol (>1 µM), multiple important pathways may be affected. For example, at 2 µM, Celastrol starts to downregulate phospho-AKT473. Therefore, caution should be taken in terms of the Celastrol’s concentration/dosage used for *in vitro* or *in vivo* study. As shown in Figure 2B, lower concentration of Celastrol such as 0.05 µM and 0.1 µM, dramatically decreased p536 expression but without affecting AR and ERG expression, indicating there may be other factors involved in regulating p536 expression when cells are exposed to very low concentration of Celastrol. These factors include several kinases such as IKKβ, IKKε and NAK (NF-KappaB activating kinase) which have been reported to be able to phosphorylate Ser536 in different systems [42], [43], [44], [45], [46] and other factors such as JNK and p53 [47]. Analysis of these factors should be included in future studies. Interestingly, we also observed that Celastrol can affect AR3 expression, a major AR active isoform, which plays an important role in promoting androgen-independent growth of PCa cells and is expressed higher in castration resistant PCa [48]. Studies showed that although AR3 regulates genes in common with full length AR, other targets may be unique to AR or to AR3 [48]. Therefore, when designing therapy to inhibit AR signaling, the existence of these active AR variants needs to be taken into consideration. Unfortunately, we were not able to verify AR3 inhibition by Celastrol *in vivo* by western blot due to insufficient tumor mass collected. Larger animal studies are clearly needed to verify this observation regarding Celastrol’s therapeutic potential.

Studies have shown Celastrol can inhibit PCa tumor growth *in vivo* [15], [22], [23], [35] using PC3 and C4-2B but never VCaP cells. Our *in vitro* data demonstrates that VCaP cells are more sensitive to Celastrol treatment than LNCaP, PC3 and DU145 cells. Since LNCaP expresses AR (PC3/DU145 are androgen independent cells), the comparison of drug response between VCaP and LNCaP will be more relevant. VCaP cells express high level of p536, ERG and AR as well as easily detectable amounts of AR3. Based on different properties of cells and the fact that Celastrol can significantly target AR-ERG-NF-κB signal cascade which are abundant in VCaP cells, we predict VCaP would be more sensitive to Celastrol treatment in animals than LNCaP. Whether this is true *in vivo* requires further investigation. Almost all prior *in vivo* studies used 1–4 mg/kg of Celastrol [15], [22], [35], [39]. Since we observed certain weight loss (10–25%) in mice when treated with 1 mg/kg/d for 16 times in our preliminary studies, we optimized our treatment condition to 0.5 mg/kg for 4 times a week. Under such condition, there was still 2–7% weight loss by the end of experiment in the treatment group but the difference is not significant between treated and untreated mice, and these animals showed no decreased activity or anorexia. We do not know the exact cause for the discrepancy we observed in 1 mg/kg/d Celastrol treated animals with other previous reports in terms of weight loss [15], [35]. One possible reason could be the mouse strain, age, or the initial weight of the mice. We found that mice whose weight were more than 30 g before the Celastrol treatment handled the treatment much better than the smaller ones. Our data showed significant reduction of tumor growth by 0.5 mg/kg Celastrol with no obvious adverse effects, suggesting the effective plasma levels of Celastrol were reached. Studies have shown that Celastrol concentration in plasma and tissues can be measured using HPLC or LC-MS/MS methods [49], [50]. However, no complete analysis of Celastrol’s pharmacokinetic/pharmacodynamic profile has been done in mice, rats or humans [15], [16]. Therefore, more detailed toxicological studies are needed to further document the effective dosage of Celastrol for live animals before its potential clinical trial in humans. We confirmed that 0.5 mg/kg Celastrol can significantly target AR-ERG-p536 expression *in vivo*. Whether even lower doses would show similar effects in VCaP tumor growth in mice needs to be tested. In addition, Celastrol has also been found to be bioavailable through oral administration in PC3 xenograft model [35]. We can also test this in our VCaP xenograft models in the future. The information about bioavailability of Celastrol will expedite its potential path to clinical trials.

In summary, we have shown that Celastrol has potent inhibitory effects on T/E fusion expressing VCaP cell growth both *in vitro* and *in vivo*. The fact that Celastrol targets AR-ERG-NF-κB signaling and inhibits human VCaP PCa tumor growth *in vivo* provides strong support for the proof-of-concept of using Celastrol for T/E fusion expressing PCa, which may benefit more than half of PCa patients who carry T/E fusions in their tumors.
